# Microbes in the Era of Circadian Medicine

**DOI:** 10.3389/fcimb.2020.00030

**Published:** 2020-02-05

**Authors:** Claudio Costantini, Giorgia Renga, Federica Sellitto, Monica Borghi, Claudia Stincardini, Marilena Pariano, Teresa Zelante, Flavia Chiarotti, Andrea Bartoli, Paolo Mosci, Luigina Romani, Stefano Brancorsini, Marina Maria Bellet

**Affiliations:** ^1^Department of Experimental Medicine, University of Perugia, Perugia, Italy; ^2^Reference Centre for Behavioural Sciences and Mental Health, Istituto Superiore di Sanità, Rome, Italy; ^3^Department of Veterinary Medicine, University of Perugia, Perugia, Italy

**Keywords:** circadian rhythm, bacteria, fungi, host, infections, immune system

## Abstract

The organisms of most domains of life have adapted to circadian changes of the environment and regulate their behavior and physiology accordingly. A particular case of such paradigm is represented by some types of host-pathogen interaction during infection. Indeed, not only some hosts and pathogens are each endowed with their own circadian clock, but they are also influenced by the circadian changes of the other with profound consequences on the outcome of the infection. It comes that daily fluctuations in the availability of resources and the nature of the immune response, coupled with circadian changes of the pathogen, may influence microbial virulence, level of colonization and damage to the host, and alter the equilibrium between commensal and invading microorganisms. In the present review, we discuss the potential relevance of circadian rhythms in human bacterial and fungal pathogens, and the consequences of circadian changes of the host immune system and microbiome on the onset and development of infection. By looking from the perspective of the interplay between host and microbes circadian rhythms, these concepts are expected to change the way we approach human infections, not only by predicting the outcome of the host-pathogen interaction, but also by indicating the best time for intervention to potentiate the anti-microbial activities of the immune system and to weaken the pathogen when its susceptibility is higher.

## Introduction

The organisms from most kingdoms of life have evolved in an environment characterized by circadian cycles of light and dark imposed by the planet's rotation around its own axis, and have adapted to anticipate these environmental changes and regulate their behavior and physiology accordingly (Bell-Pedersen et al., 2005). The evolution of an intrinsic circadian clock at the basis of such environmental-induced physiological adaptation has been the subject of intense research and fundamental aspects, conserved but with specific differences, have been identified in various kingdoms (Bell-Pedersen et al., 2005). In higher organisms, including mammals, this endogenous clock regulates all aspects of physiology, including sleep-wake cycle, locomotor activity, feeding behavior, body temperature, hormonal and enzymatic functions.

**Figure 1:**
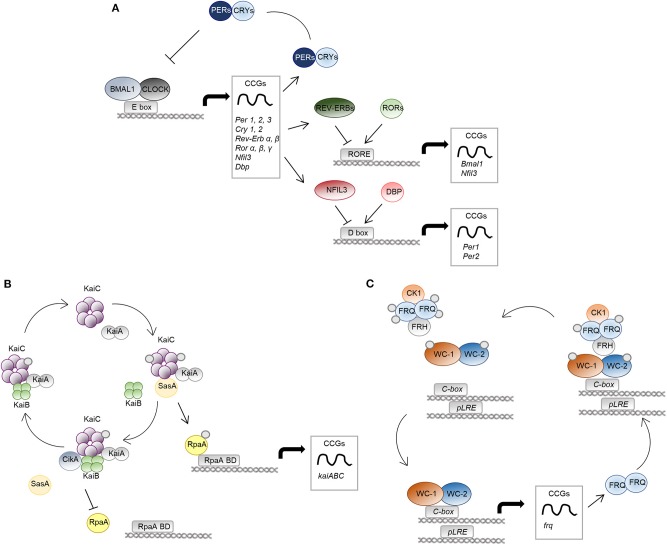
The molecular clock in mammals, bacteria and fungi. The molecular mechanisms of the circadian clock in mammalian cells **(A)**, *S. elongatus* **(B)**, and *N. crassa* **(C)** are schematically represented. **(A)** The positive regulators CLOCK-BMAL1 activate transcription of genes with E-box elements in their promoters, named clock-controlled genes (CCGs). Among them, there are regulators of the negative limb (PER and CRY proteins) as well as other transcription factors, such as REV-ERBs/RORs and DBP/NFIL3, controlling the expression of genes with RORE and D-box DNA-response elements in their promoter, respectively. **(B)**, KaiA binds KaiC hexamers and promotes its phosphorylation. The protein SasA competes with KaiB for the binding to phosphorylated KaiC. SasA binding induces activation of the output protein RpaA, controlling global gene expression through RpaA DNA binding domains (RpaA BD). Accumulation of KaiB and binding to KaiC causes inactivation of KaiA, leading to KaiC dephosphorylation and inactivation. Moreover, CikA interacting with KaiB cause dephosphorylation and inactivation of RpaA. **(C)** The positive elements of the WCC complex (WC-1 and WC-2) control the circadian expression of the frequency (*frq*) gene, the negative arm of the feedback loop. FRQ homodimers associate to the protein FRH and to casein kinase 1 (CK1), causing phosphorylation and inactivation of WCC. Progressive phosphorylation of FRQ leads to dissociation from the WCC complex and proteasomal degradation.

A circadian pacemaker in the hypothalamic suprachiasmatic nucleus receives environmental signals and coordinates the oscillating activity of clocks located in peripheral tissues. At the molecular level, the clock machinery is based on a transcriptional-translational feedback autoregulatory loop, in which transcriptional activators induce the expression of repressor genes and of many tissue-specific clock-controlled genes (CCGs) (Partch et al., 2014). At the core of the mammalian circadian clock are two basic helix-loop-helix-PER-ARNT-SIM transcription factors, CLOCK and BMAL1 (C/B), whose heterodimers drive the rhythmic expression of repressor genes, including Period (Per) and Cryptochrome (Cry) genes, through binding to E-box elements within the promoter of these genes. The resultant PER/CRY protein complexes inhibit C/B-mediated transcription, completing the negative feedback loop essential for clockwork function (Sangoram et al., 1998; Griffin et al., 1999; Kume et al., 1999). C/B also induce the nuclear receptors Rev-Erbα and Rev-Erbβ, which accounts for BMAL1 rhythmic expression together with RORα. Rev-Erbs and RORα, respectively, repress and activate *Bmal1* gene expression in a time-specific manner, through competitive binding to the same RORE DNA-binding sites, thereby constituting a second important interlocking feedback loop (Preitner et al., 2002; Sato et al., 2004). A third regulatory loop is the one that includes transcription factors like DBP and NFIL3 that, respectively, activate and repress core clock genes, such as *Per1*, containing D-box elements in its promoter (Yamaguchi et al., 2000; Mitsui et al., 2001). All these interlocked loops also control the circadian profile of expression of many CCGs not directly involved in this core mechanism (Figure 1A). Furthermore, post-translational regulation and epigenetic mechanisms add additional levels of complexity to the system (Bellet and Sassone-Corsi, 2010), ultimately leading to the modulation of the expression of a large array of cellular transcripts in each tissue, corresponding to nearly half of the genes in the mouse genome if we consider multiple tissues, with profound consequences in the majority fundamental biological processes (Zhang et al., 2014). As such, perturbations of this sophisticated mechanism inevitably result in pathological conditions, as demonstrated by both animal studies and epidemiological data in people undergoing abnormal lighting schedule exposure (i.e., shift workers), in which an increased risk of several type of diseases, from depression to metabolic disorders, to cardiovascular and inflammatory diseases to cancer, has been documented (Bass and Lazar, 2016). It comes that our understanding of disease onset and progression as well as our approach to disease treatment cannot be separated from a deeper understanding of the circadian rhythms at their basis. Specifically, the molecular clock controls fundamental aspects of the immune response, by regulating the expression and function of immune modulators and immune cells, and its dysregulation may lead to inflammatory diseases, immunodeficiency and augmented risk of infections (Curtis et al., 2014). Moreover, the importance of circadian rhythms in infectious diseases is 2-fold, functioning at the levels of both host and pathogen.

Herein, starting from this last concept, we summarize the current evidence for the presence of circadian rhythms in pathogenic bacteria and fungi, discuss the role of the circadian clock in controlling antibacterial and antifungal host defense, and speculate on the impact of the application of the circadian medicine in infectious diseases.

## An Endogenous Clock in Microbes

Three rhythmic properties are considered reliable and solid proof of the presence of a circadian clock in every organism (Bell-Pedersen et al., 2005). The first one is the presence of free-running rhythms, defined as self-sustained oscillations with a 24-h period that persist in constant conditions (i.e., constant darkness in nocturnal, constant bright in diurnal animals). Another essential property is temperature compensation, referring to the stability and persistence of a free-running rhythm within a range of physiological temperatures. Lastly, the entrainment, which is the synchronization of a rhythm by an environmental “time giver” called zeitgeber (ZT) (i.e., light, temperature). In nature, there are countless examples of organisms with all these three properties, including most eukaryotic (fungi, protozoa, plants, birds, and mammals) and some prokaryotic organisms (photosynthetic bacteria). In this section, we discuss the current knowledge on the presence of circadian rhythms in bacteria and fungi, particularly focusing in those important for human health, and the potential molecular components driving these rhythms.

### Circadian Rhythm in Non-photosynthetic Bacteria

Among prokaryotes, the most well-described and extensively studied circadian rhythm is that of the cyanobacteria *Synechococcus elongatus*, which became a model organism for studies on the circadian field (Lakin-Thomas and Brody, 2004; Bell-Pedersen et al., 2005). Here, three core clock genes, named *kaiA, kaiB*, and *kaiC*, encode for proteins essential for rhythmic regulation of almost the entire genome and for the timing of cellular events, including cell division, photosynthesis, and amino-acid uptake, ultimately leading to enhanced fitness and to a selective advantage (Kondo et al., 1994). Unlike eukaryotes, they exclusively act at the post-transcriptional level (Nakajima et al., 2005; Tomita et al., 2005) through a mechanism involving formation of multi-protein complexes, events of phosphorylation and dephosphorylation, and conformational changes in the core biochemical oscillator (Swan et al., 2018). Briefly, the central loop involves the activation of KaiA during the day through interaction with proteins sensing environmental information, such as light, called Circadian input kinases (Cik). Once activated, KaiA interacts with the ATPase KaiC, and stimulates its autophosphorylation and oscillations between different quaternary structures. Moreover, phosphorylated KaiC binds to factors involved in the activation of rhythmic gene expression, such as SasA and RpaA. The cycle ends when KaiB binds KaiC and inhibits KaiA-stimulated phosphorylation of KaiC (Figure 1B; Johnson et al., 2017).

Some reports described the presence of rhythmic processes in other photosynthetic bacteria, such as the purple bacteria *Rhodospirillum rubrum* and *Rhodobacter sphaeroides* (Van Praag et al., 2000; Min et al., 2005). Although not strictly following the definition of circadian rhythms, the identification of these rhythms strengthens the hypothesis that more prokaryotes can possess an intrinsic cell-autonomous timing mechanism. However, if a circadian clock exists in non-photosynthetic bacteria, particularly those important for human health, and how it regulates processes essential for bacteria survival and adaptation to environmental changes is still an open question (Sartor et al., 2019).

There are only few examples describing the presence in prokaryotes of endogenous oscillations that meet the classical definition of circadian rhythms (Table 1). This evidence dates back to the last century, when growth rhythms were described in proteobacteria, including *Escherichia coli* (Rogers and Greenbank, 1930) and *Klebsiella pneumoniae* cultures (Sturtevant, 1973). However, these rhythms were not temperature compensated, therefore not classically definable circadian. More recently, a circadian oscillation was reported in the growth of the soil bacteria *Pseudomonas putida* (Soriano et al., 2010), as well as in *Bacillus subtilis* (Sartor et al., 2019), a famous gram-positive bacterium, important for its various industrial and medical applications. This bacterium in particular displays a 24-h oscillation in gene expression associated with growth, differentiation and spore formation, and it has been pointed out as a potential model organism for further circadian studies in non-photosynthetic bacteria.

**Table 1:** Regulation of host and/or microbes circadian rhythms in selected bacterial and fungal infections.

**Bacterial/Fungal species relevant for human health**	**Evidences of circadian rhythm/circadian components**	**Evidences of time-dependent host response**	**Reference**
*S*. Thyphimurium		Enhanced clearance at night (ZT16), clock-dependent	Bellet et al., 2013
*L. monocytogenes*		Enhanced clearance at ZT8, dependent on BMAL1 in Ly6C^hi^ monocytes	Nguyen et al., 2013
*S. pneumoniae*		Enhanced clearance at ZT12, dependent on BMAL1 in Clara cells	Gibbs et al., 2014
*C. trachomatis*		Enhanced clearance at ZT15	Lundy et al., 2019
*H. pylori*		Enhanced lymphocyte migration to lymph nodes at ZT7, circadian clock-dependent	Druzd et al., 2017
*E. coli*	Growth rhythms, receptors for blue light		Rogers and Greenbank, 1930; Gomelsky and Klug, 2002
*P. aeruginosa*	Receptors for red light		Davis et al., 1999
*L. pneumophila*	*kai*B and *kai*C-encoding genes		Loza-Correa et al., 2010
*E. aerogenes*	Swarming and motility rhythms		Paulose et al., 2016
*A. fumigatus*	*wc*-1 and *wc*-2-encoding genes	Time-dependent clearance	Salichos and Rokas, 2010; Chen et al., 2018
*C. albicans*		Enhanced clearance at ZT13	Adrover et al., 2019

Examples of alleged circadian components in these organisms, identified by studying gene homology, are instead more frequent. Homologs of *kai* genes (*kaiC* in particular) have been found in various phyla within the eubacteria reign, including proteobacteria and bacteroidetes (Loza-Correa et al., 2010), like the root-associated bacteria forming plant symbionts *Sinorhizobium medicae* and *Pseudomonas putida*. Moreover, in addition to kai homologs, other proteins in bacteria that might work as clock components are peroxiredoxin, a class of proteins whose redox state is rhythmically and autonomously oscillating in cyanobacteria and archea. Peroxiredoxin proteins have been found in almost all species of bacteria, thus suggesting that their redox rhythm might be conserved and act as the timekeeper in these organisms (Edgar et al., 2012). Moreover, different types of photoreceptors have been found in many non-photosynthetic species. Between them, some phytochrome-like photoreceptors, sensing red light, are present in various plant symbionts. Furthermore, several blue light sensing photoreceptors were found in bacteria forming association with plant roots, like *B. subtilis* (Losi et al., 2002).

If we specifically focused on bacteria important for human health, there is still limited evidence in favor of the presence of a circadian clock in these species. *Legionella pneumophila*, an environmental bacterium and opportunistic human pathogen causing severe pneumonia, contains genes encoding KaiB and KaiC, but it is not known whether their presence provides advantages in terms of adaptation to the environment, replication and virulence (Loza-Correa et al., 2010). Similarly, *Pseudomonas aeruginosa* or *E. coli* contain red and blue-light photoreceptors, respectively, that might be associated to adaptation to day-night cycles (Davis et al., 1999; Gomelsky and Klug, 2002). In the gut, the commensal bacterium *Enterobacter aerogenes* expresses a circadian rhythm in swarming and motility, which is enhanced in the presence of melatonin (Paulose et al., 2016). To date, this last example appears to be the most clear demonstration of a 24-h rhythm in a non-cyanobacterial prokaryote. An observation suggesting the possibility that the circadian clock in the host, through the regulation of intestinal functions, gene expression and hormones like melatonin, can both modulate the microbiota as a community, as discussed below, but also through the entrainment of single bacterial clocks eventually present.

In conclusion, we still have very poor knowledge on the presence of circadian rhythms in prokaryotes other than cyanobacteria. It is hard to imagine that organisms like bacteria have not evolved a mechanism to deal with environmental changes dictated by the day/night cycle and adapt to them. Whether our lack of knowledge is due to assessment of rhythm being conducted in the wrong experimental conditions, investigations by using different condition of growth or maintenance might provide relevant advances. Moreover, the complexity of the communities in which bacteria are used to be found needs to be taken into consideration. It is tempting to speculate that significant aspects of the circadian physiology of prokaryotes still have to be unveiled and will provide us novel exciting information that might be relevant for human pathophysiology.

### Circadian Rhythms in Fungi

Among fungi, the ascomycete *Neurospora crassa* is the only model system in which the presence of a well-defined molecular clock has been systematically studied and documented. Along with *Drosophila melanogaster, N. crassa* has represented a model organism for the study of the circadian rhythms and has been instrumental to decipher the molecular mechanisms underlying its functioning (Loros, 2019). The current model of the circadian rhythm in *N. crassa* identifies in the White Collar Complex (WCC), a heterodimer formed by the two transcription factors White Collar 1 (WC-1) and White Collar 2 (WC-2), the basis for the Transcriptional Translational Feedback Loop (TTFL) (Larrondo and Canessa, 2019). Indeed, WCC regulates the expression of the *frequency* (*frq*) gene, which occurs by two distinct modalities. Under constant darkness, WCC binds to the *clock-box* (*c-box*) and mediates the circadian expression of *frq*. Alternatively, in the presence of light, which is sensed by the light-, oxygen- and voltage-sensing (LOV) domain of WC-1, multimers of WCC are formed by LOV-LOV interactions that shift the specificity to the *proximal light regulatory element* (*pLRE*), responsible for the light-dependent increase in *frq* expression (Larrondo and Canessa, 2019). The FREQUENCY (FRQ) protein then associates with FRQ-RNA-Helicase (FRH) and casein kinase 1 (CK1) to form the FFC complex that, in turn, phosphorylates WC-1 and WC-2 to abolish their DNA binding ability (Wang et al., 2019). At the same time, FRQ becomes progressively phosphorylated and dissociates from WCC, thus allowing WCC to recover its function and start a new cycle (Figure 1C; Larrondo and Canessa, 2019).

While an extensive knowledge has been accumulated on the circadian clock in *N. crassa*, studies on other fungi has remained very limited (Larrondo and Canessa, 2019). The presence of the *frq* gene has been demonstrated in other *Neurospora* species (Lewis and Feldman, 1996) and initial phylogenetic studies have revealed the presence of the *frq* gene in other species within the family *Sordariaceae*, to which *N. crassa* belongs (Merrow and Dunlap, 1994). Interestingly, a functional complementation was observed upon inserting the *Sordaria fimicola frq* gene in a *N. crassa frq* null strain (Merrow and Dunlap, 1994). In addition, the presence of the *frq* gene was shown in two species from other families within the ascomycete fungi, namely *Chromocrea spinulosa* and *Leptospheria australensis* (Lewis and Feldman, 1996; Lewis et al., 1997), with distinct abilities to complement a conditionally arrhythmic *Neurospora* mutant (Lewis et al., 1997). However, whether the other components *wc-1* and *wc-2* were similarly present was not investigated, thus limiting the possibility to conclude that a molecular clock, similar to that described in *N. crassa*, is likely functioning in these species.

As soon as more fungal genomes became available, a more extensive genetic analysis has been performed to include not only the *frq* gene, but also the other components *wc-1* and *wc-2* of the oscillator. One such analysis on 17 full fungal genomes has revealed that *Gibberella zeae, Magnaporthe grisea* and *Podospora anserina* contained all the genes identified in *N. crassa* for a functional oscillator (Lombardi and Brody, 2005). Of note, some species, including *Aspergillus nidulans*, did not appear to contain the *frq* gene, although *wc* homologs were present (Lombardi and Brody, 2005). This finding is of interest since the existence of an entrainable temperature-compensated rhythm of sclerotia formation has been described in *Aspergillus flavus* (Greene et al., 2003) and an enzyme rhythm was also shown in *Aspergillus nidulans* (Greene et al., 2003), suggesting that the presence of a *frq* gene ortholog may not be required for a circadian feedback. A subsequent analysis on 42 fungal genomes has shown that a complete FRQ-WCC system is universally seen among the Sordariaceae while WC-1 is strongly conserved throughout the fungi, including strains lacking FRQ (Dunlap and Loros, 2006). A more recent analysis on 64 fungal proteomes established the gain of FRQ within the Ascomycetes in the classes Sordariomycetes, Leotiomyecetes, and Dothideomyecetes while WC-1 and WC-2 were present not only in other classes of Ascomycetes, such as Eurotiomycetes, that includes, among others, Aspergillus species, but also in other phyla, such as Basidiomycetes and Zygomycetes (Salichos and Rokas, 2010). WC-1 and WC-2, however, were lost in the class of Saccharomycetes, implying that, among others, Candida species do not express any of the FRQ, WC-1, and WC-2 clock components (Salichos and Rokas, 2010). The time of appearance of the *frq* gene has been disputed in subsequent studies. Indeed, an FRQ homolog was first identified in *Pyronema confluens*, an ancestor of filamentous ascomycetes, thus pointing to an earlier appearance of the *frq* gene (Traeger et al., 2013) that was further anticipated in a subsequent analysis of 473 fungal genomes in which an FRQ-like sequence was identified in the early diverging ascomycete *Saitoella complicata* (Montenegro-Montero et al., 2015). According to a recent study, FRQ sequences were also identified in Basidiomycota, Zoopagomycota, and Mucoromycotina (Brody, 2019).

All in all, these results indicate that, with the notable exception of *N. crassa*, the knowledge on the molecular mechanisms regulating the circadian rhythms in fungi is scarce. The prototypical FRQ-WCC oscillator do not appear to be universally present in fungi, despite the presence of *bona fide* circadian rhythms in some strains, thus raising the possibility that mechanisms other that FRQ-WCC might participate in their regulation. As discussed in detail below, the plant pathogen *Botrytis cinerea* is the only fungal pathogen in which a molecular circadian clock has been identified and characterized (Hevia et al., 2015). At present, it is unclear whether and how a circadian clock is organized in common human fungal pathogens, such as *A. fumigatus* and *C. albicans*. Indeed, the former expresses *wc-1* and *wc-2* homologs, but not *frq*, while the latter does not express any of these components. Whether a clock system might exist in these organisms and its potential relevance for the acquisition of virulence is still an almost unexplored field, thus opening up novel areas of investigation for therapy (Hevia et al., 2016).

## Circadian Control of the Host Response to Microbial Infections

The mammalian circadian clock has been reported to control the expression and function of immune modulators and immune cells (Curtis et al., 2014). From an adaptive point of view, a major purpose of such circadian modulation might be to anticipate daily changes in activity and feeding and the associated risk of infections. Accordingly, studies in rodents have demonstrated a circadian regulation of host defense reactions against specific pathogens, as we will discuss below. What has emerged from these studies is that the rhythmic environment that a microbe finds inside its host is able to influence microbial virulence, level of colonization and tissue damage and can confer a competitive advantage of some species in specific moments of the day. Here, we summarize the mechanisms of circadian regulation of immune functions that most significantly impact on the outcome of bacterial and fungal infections, during which a circadian regulation of the host response have been demonstrated.

### Circadian Effects on Immune Functions

The last two decades have provided clear evidence of the presence of a daily rhythm in many immune functions (see Scheiermann et al. for an extensive recent review on clocks and the immune system; Scheiermann et al., 2018). Almost all innate immune cells possess an intrinsic core molecular clockwork that governs multiple functions, including phagocytic activity, production of cytokines, chemokines and cytotoxic factors in specific cell types (Arjona and Sarkar, 2005; Keller et al., 2009; Gibbs et al., 2012; Silver et al., 2012a; Nguyen et al., 2013). Moreover, the expression of some pattern recognition receptors (PRR) is regulated in a circadian manner. For example, different types of Toll-like receptors (*Tlr*), including *Tlr1-5* and *Tlr9*, as well as that of NOD-like receptor *Nod2*, are rhythmically activated by RORα in intestinal epithelial cells at the transcriptional level, while the NOD-like receptor family pyrin domain containing 3 gene (*Nlrp3*) displays diurnal rhythm in liver and colon and is inhibited by Rev-Erbα (Mukherji et al., 2013; Wang et al., 2018). The circadian clock also controls trafficking, localization and turnover of innate immune cells, both at steady-state conditions and during infections (Scheiermann et al., 2012; Casanova-Acebes et al., 2013; Gibbs et al., 2014; Haspel et al., 2014). This results in circadian variations in the relative abundance and distribution of immune cell types between bone marrow, lymphoid organs, the bloodstream and specific sites of infection, thus clearly affecting the host response to pathogens, as discussed below.

Adaptive immune response is also regulated by the clock system mainly in terms of lymphocyte differentiation, polarization and trafficking. For example, the clock system, through Rev-Erbα and Nfil3, controls T cell polarization in T helper 17 (T_H_17) cells. This is through Nfil3 rhythmically suppressing the production of Rorγt, a transcription factor essential for T_H_17 differentiation (Yu et al., 2013). This regulation is particularly relevant for mucosal immunity against bacterial and fungal pathogens in the lung and the intestine, since these cells classically produce IL-17 and IL-22, two cytokines mediating important mechanisms of resistance which are essential for the initial control of microbial burden, but whose over-activation might lead to detrimental persistent inflammation. A diurnal change in the proportion of T_H_17 cells might render animals more or less susceptible to infections, by influencing the balance between resistance and tolerance at specific times of the day. The circadian timing is also important in the interaction between T cells and antigen-presenting cells (APC) (Fortier et al., 2011). Importantly, the rhythmic expression of PRR in the APC, together with the circadian variation in the number of immune cells in lymphoid organs is likely to dictate a rhythm for both humoral and cellular adaptive immune response, with important consequences for improving vaccination efficacy, as recent studies in both mice and humans have shown (Long et al., 2016; Suzuki et al., 2016; Druzd et al., 2017). Here we discuss the main findings describing how the circadian clock controls host response during bacterial and fungal infections.

### The Host Immune Clock Activity During Bacterial Infections

A circadian response to administration of lethal doses of the bacterial product lipopolysaccharide (LPS) in mice was recognized for the first time more than 50 years ago (Halberg et al., 1960). After this pioneering study, many models of bacterial infection have been tested and revealed how the host response profoundly change its nature, magnitude and efficiency depending on the time of first contact with the microorganism (Table 1). In some of these studies, highlighted below, a definition of the molecular mechanisms that connect specific clock proteins to immune response has been unveiled and provided novel important understandings that might be useful to improve potential chronotherapeutic applications.

#### Microbial Products (Pathogen Associated Molecular Pattern, PAMP) Circadian Effects

Halberg et al. in 1960 observed a dramatic increase in lethality upon LPS-induced endotoxic shock following the administration of LPS performed in the evening, corresponding to the onset of activity in mice (Halberg et al., 1960). It was subsequently revealed that this time correlates with enhanced induction of pro-inflammatory cytokines and recruitment of leukocytes into tissues (Gibbs et al., 2012; Scheiermann et al., 2012). Disruption of the circadian rhythm worsens this response, as LPS administration in mice following induction of experimentally induced chronic jet lag produced a more severe endotoxemia compared to controls, with severe hypothermia and increased mortality (Castanon-Cervantes et al., 2010). Furthermore, challenge of cells *in vitro* with LPS or lipoteichoic acid at different ZT or CTs is associated with rhythmic production of proinflammatory cytokines, an effect dependent on the presence of a functional clock within each specific cell (Liu et al., 2006; Keller et al., 2009; Bellet et al., 2013; Heipertz et al., 2018). Similarly, aerosol LPS administration in C57BL/6 mice across the circadian cycle produced a different pro-inflammatory response, with enhanced production of inflammatory cytokines and number of inflammatory cells in broncoalveolar lavage (BAL) at CT0 compared to other CTs (Gibbs et al., 2014). Besides LPS, recent studies revealed a time-dependent response of innate immune cells to stimulation with different type of PAMPs, in terms of circadian rhythmicity of both cytokine expression and phagocytosis activity (Hayashi et al., 2007; Ella et al., 2016; Silver et al., 2018).

#### Sepsis Models

Sepsis is a life-threatening syndrome occurring because of a dysregulation of the host response to infection leading to severe organ injury, septic shock, and high risk of mortality. Circadian susceptibility to septic shock has been investigated in numerous works. Initially focused on survival differences between CTs, some of these first reports demonstrated a circadian rhythmicity in the resistance to subcutaneous infection with *Streptococcus pneumoniae* (Feigin et al., 1969; Shackelford and Feigin, 1973). Subsequent studies obtained results suggesting a circadian variation in the response to sepsis induced after cecal ligation and puncture (CLP), the differences being variably associated to a TLR2 or TLR9-dependent mechanism (Silver et al., 2012b; Heipertz et al., 2018), to the control of immune checkpoint pathways by BMAL1 (Deng et al., 2018) and to neutrophils mobilization and aging (Adrover et al., 2019). Remarkably, recent human pilot studies revealed that blue light stimulation would confer protection in mice during CLP-induced sepsis, as well as during lung infection with *Klebsiella pneumoniae*, Indeed, blue light stimulation seems to reduce bacterial growth, dissemination and inflammatory response, ultimately improving survival, an effect that has been associated to induction of Rev-Erbα (Lewis et al., 2018; Griepentrog et al., 2019). These observations are of particular interest, as they might foster approaches aimed at improving the recovery of patients from sepsis by simple measures, such as reducing nocturnal light, improving quality of sleep or apply blue-spectrum lighting during the perioperative period in intensive care units (Hrushesky and Wood, 1997; Herdegen, 2002).

#### *Salmonella Thyphimurium* and Lipocalin-2

*Salmonella enterica* serovar Thyphimurium is a foodborne pathogen responsible of gastrointestinal infections, most frequently through contaminated water or food. In a mouse model of infectious colitis with this pathogen, it was shown that animals exhibited a time-specific response to infection (Bellet et al., 2013). Indeed, the clearance of the bacteria was enhanced at night (ZT16), while the degree of inflammation in the cecum was higher when mice were infected in the morning (ZT4). This response appeared to require a functional clock, as it was absent in *Clock* mutant mice. Importantly, transcriptome analysis of cecum of these mice 72 h post-infection revealed that, among others, genes encoding antimicrobial proteins displayed a robust circadian oscillation. Salmonella is resistant to many antimicrobial proteins secreted in the inflamed gut, thus explaining why its growth is enhanced and not limited by intestinal inflammation. In particular, the antimicrobial peptide lipocalin-2, which acts as a bacteriostatic factor by sequestering iron as a nutrient for susceptible bacteria, is higher during day than at night. This allows for the outgrowth of Salmonella, which is resistant to lipocalin-2 through the synthesis of the iron chelator salmochelin, over susceptible bacteria of the resident microbiota and contributes to explain the increased colonization at that time of the day.

#### *Listeria monocytogenes* and Ly6C^hi^ Monocytes

The foodborne pathogen *Listeria monocytogenes* is a Gram-positive intracellular bacterium causing listeriosis in both animals and humans. While *L. monocytogenes* infection in humans is not frequent, the infection and mortality become elevated in high-risk populations, such as immunocompromised individuals. Typically, a monocyte-mediated immune defense against this pathogen controls bacterial clearance at sites of infection, particularly liver and spleen. In a mouse model of infection with L*. monocytogenes* (Nguyen et al., 2013), it was shown that mice infected intraperitoneally at ZT0 displayed a higher colonization in spleen, liver and peritoneum, compared to ZT8. This was associated with a diurnal rhythm of Ly6C^hi^ inflammatory monocytes, whose recruitment was increased at ZT8, together with an enhanced inflammatory response at the same time point. Genetic disruption of the clock machinery in myeloid cells, obtained by knocking-out *Bmal1* specifically in these cells, abolished this phenotype. Remarkably, the same study showed that BMAL1 plays a central role in regulating the oscillation of Ly6C^hi^ inflammatory monocytes. Indeed, BMAL1, together with CLOCK, is recruited to the promoter of chemokines like *Ccl2, Ccl8*, and *s100a8*, essential drivers of monocytes recruitment, and represses their expression in a time-specific manner, through binding and recruitment of histone methyltransferases of the polycomb repressive complex 2 (PRC2).

#### *Streptococcus pneumoniae* and CXCL5

Gibbs et al. reported a time-of-day variation in the host response to *S. pneumoniae* pulmonary bacterial infection (Gibbs et al., 2014). In particular, an increased recruitment of neutrophils was observed at ZT12 compared to ZT0, corresponding to a subsequent reduction of bacterial burden and dissemination at the same time point. This response appeared not to have been influenced by the integrity of the circadian clock in monocyte or neutrophils, but it was rather associated with the function of the clock system in bronchiolar epithelial cells named Clara cells. Indeed, Clara cells *Bmal1*-null mice lack the circadian gating in the host response to *S. pneumonia* that drives the circadian recruitment of neutrophils to the lung, thus influencing the outcome of the infection. Gibbs et al. elegantly showed how the circadian clock controls the rhythmic expression of the neutrophil chemoattractant protein CXCL5 in these cells. They demonstrated that the rhythmic regulation of *Cxcl5* gene expression relies on glucocorticoid receptor occupancy at the promoter of the gene. The recruitment is absent in mice with ablation of *Bmal1* in Clara cells, despite a conserved rhythmic corticosteroid signaling, an information particularly relevant because it suggests that the efficacy of dexamethasone treatment is strongly dependent on the presence of a functional clock within a specific tissue or a specific cell-type within a tissue.

#### *Chlamydia* spp. Genital Infection

Genital chlamydia infection is one of the most common bacterial sexually transmitted infections, leading to a range of manifestations, such as pelvic inflammatory disease, cervicitis and salpingitis and infertility. A recent report showed how intravaginal infection of mice with different strains of *Chlamydia* resulted in increased colonization, increased pro-inflammatory response and increased incidence of histopathological signs of disease (inflammation, hyperplasia, presence of cysts) when the infection was performed at ZT3 compared to ZT15 (Lundy et al., 2019). Moreover, they observed that time of day of infection also affected the fertility rate following repeated infections, thus suggesting that clinical differences in the susceptibility to develop symptomatic infections and long-term complications might be associated to a different time of exposure to the pathogen.

#### *Helicobacter pylori* and Adaptive Immune Response

By using a model of chronic intra-gastric infection with *Helicobacter pylori*, Druzd et al. recently demonstrated that adaptive immune responses to pathogens shows circadian rhythmicity. Indeed, they demonstrated a circadian rhythm in lymphocytes migration to lymph nodes (Druzd et al., 2017), with a peak of entrance at night, and exit in the lymph during the day. This rhythmic trafficking is driven by the endogenous clock in lymphocytes controlling pro-migratory factors, such as the chemokine receptors CCR7 and the receptor for the chemoattractant phospholipid sphingosine-1-phosphate. Genetic disruption of the circadian clock in T cells abolishes this rhythmicity. Mice infected at different ZT displayed, 3 weeks following infection, a significantly higher number of cells within lymph nodes at ZT7 compared to other times, thus proving the relevance that such regulation might have in mounting an efficient adaptive immune response against pathogens.

### The Diurnal Host Response to Fungal Infections

Fungi are associated to a broad spectrum of pulmonary, cutaneous or gastrointestinal diseases in mammals, whose clinical relevance has grown because of an increasing population of immunocompromised hosts. A circadian regulation of the host response to pulmonary fungal infection has been demonstrated in mice infected with the environmental filamentous fungus *Aspergillus fumigatus* (Chen et al., 2018). Specifically, it was reported that the challenge with *A. fumigatus* in mice, at a low dose, resulted in a significantly different clearance from the lungs 1 day post-infection, depending on the time of inoculation (Chen et al., 2018). We have recently extended these results and showed that a diurnal change occurs in both level of colonization, inflammation and damage to the host in lungs of mice infected intranasally with *A. fumigatus* at medium-high doses, evaluated until 7 days post infection (M.M.B., personal communication). This time-of-day specific effect appeared to be independent from cell-intrinsic macrophage rhythms, as it was shown that transcripts and protein levels of Dectin-1, the receptor for β-glucan expressed in fungal cell walls, and phagocytosis of swollen conidia, were not under circadian control in macrophages (Chen et al., 2018). As discussed by the authors, other mechanisms involved in antimicrobial defense may be under circadian control, for instance the recruitment and/or activity of neutrophils, a major player in the innate defense against fungal infections. This observation is particularly interesting as recent reports support a role of the circadian rhythms in the regulation of neutrophil activity in homeostatic and pathogenic conditions. For instance, neutrophils infiltrate virtually every tissue in homeostatic conditions with dynamics that are specific for each tissue, the majority of which shows diurnal oscillations that are in anti-phase with those in the circulation (Casanova-Acebes et al., 2018). Notably, the diurnal pattern of neutrophil infiltration in the lung regulated the diurnal transcription of about one fourth of all the pulmonary genes under circadian control, which are involved in a variety of biological processes with potential pathological consequences (Casanova-Acebes et al., 2018). For instance, in a B16F1 melanoma model of metastasis, the diurnal infiltration of neutrophils in the lung could regulate the migration of circulating tumor cells such that the ability to form metastatic foci was dependent on the time of injection of tumoral cells (Casanova-Acebes et al., 2018). Further work of the same group have shown that a neutrophil-intrinsic circadian clock, by promoting diurnal compartmentalization of neutrophils, coordinates vascular protection and immune defense, including antimicrobial defense against fungal infection (Adrover et al., 2019). Indeed, they observed that mice were more resistant to intravenous infection with the opportunistic pathogenic yeast *Candida albicans* when the infection was performed at ZT13 compared to ZT5. Both colonization in the kidney and weight loss were reduced, while survival rate was increased, 6 days following infection at ZT13, simultaneously with an increased ratio between tissue and blood neutrophils. All in all, this information gives an initial view of the relevance that clock-regulated immune processes have in protection against fungal infections (Table 1), that might have important consequences in the management of individuals, often immunocompromised patients, undergoing fungal infections, in order to develop appropriately timed therapeutic interventions.

## Cross-Talk Between Circadian Rhythms in Host-Microbe Interactions

Given the paucity of information on circadian rhythms in bacteria and fungi, the description of cross-talks between circadian rhythms of hosts and microbes, either symbiont or pathogen, is still in its infancy. There are few examples, however, that are worth mentioning. One such example is represented by the arbuscular mycorrhizal (AM) symbiosis, which is formed by plant roots and fungi. It has been reported that the AM fungus *Rhizoglobus irregular* expresses the components of a circadian clock (Lee et al., 2018), thus raising the possibility that a cross-talk between the circadian rhythms of the AM fungus and the plant might exist. Such hypothesis has been the object of a recent review (Lee et al., 2019). As discussed by the authors, different mechanisms might be brought into play to explain a connection between the circadian rhythms of the plant and the AM fungi, including phosphorus, nitrogen, and carbon exchanges, that are under circadian control in plants and regulate mycorrhizal symbiosis (Lee et al., 2019). Another notable example is represented by the interaction between the necrotrophic plant pathogen *B. cinerea* and *Arabidopsis thaliana*, as recently reviewed (Larrondo and Canessa, 2019). In the original paper, it was demonstrated that a functional clock exists in *B. cinerea*, similar to the one described in *N. crassa*, with the ability to regulate fungal virulence, such that the outcome of the interaction between *B. cinerea* and *A. thaliana* varied with the time of the day (Hevia et al., 2015). Interestingly, although it was known that the plant defense mechanisms varied daily, it was the fungal clock that played a major role in determining the outcome of the interaction (Hevia et al., 2015). This study puts forward the interesting idea that circadian changes in the defense mechanisms of the host integrates the circadian changes in the virulence of the pathogen such that different outcomes are expected depending on the time at which the two entities interact, a paradigm that can be potentially extended to human fungal and bacterial pathogens. At present, however, this hypothesis remains unexplored.

**Figure 2:**
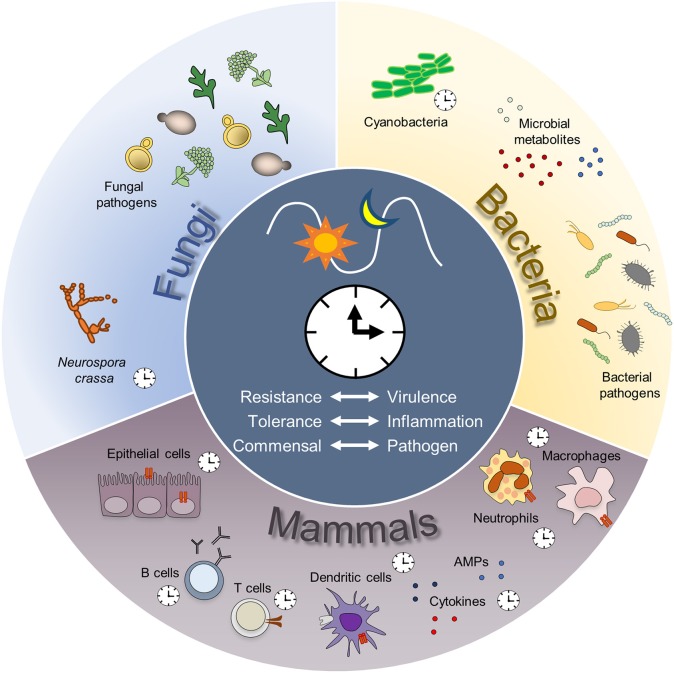
Host-microbes circadian interactions. A circadian control of antimicrobial immunity is largely documented. Endogenous circadian rhythms might exist also in bacterial and fungal species important for human health. Potential cross-talk between the clock machinery of each species might influence the balance between resistance of the host and virulence of the pathogen, leading to a time-dependent prevalence of inflammation or tolerance, ultimately defining the switch between commensal to pathogenic relationships. AMP, antimicrobial peptides.

All in all, these data indicate that a cross-talk between the circadian clocks of common human pathogens, when present, and the host may occur that regulates the outcome of their interaction (Figure 2). Indeed, although a circadian clock in human pathogens is as-yet uncharacterized, the possibility exists that a circadian rhythm regulates their activity and likely their virulence, at least in those microbes that expresses components of the prototypical circadian clock. This would interact with diurnal changes in neutrophil number, infiltration and activity, in turn associated with diurnal changes in tissue susceptibility to infection. Whether the two circadian rhythms may not only overlap, but also influence each other, remains an open area of investigation for future research.

### Circadian Rhythm and Microbial Communities

The best example so far known of cross-talk between circadian clocks in mammalian hosts and microbes is that with the gut microbiota. The relative composition and abundance of microbial species within the gut microflora is oscillating in mice and humans, the rhythmicity being driven mainly by feeding rhythms of the host (Thaiss et al., 2014; Voigt et al., 2014; Liang et al., 2015; Deaver et al., 2018). Conversely, multiple reports have shown how the gut microbiota impacts the host circadian clock and modulates circadian physiology locally and at distance (Thaiss et al., 2014; Leone et al., 2015; Murakami et al., 2016; Wang et al., 2017; Weger et al., 2019). Importantly, circadian clock disruption leads to loss of the rhythm of the microbiota, with reduction of bacterial species important for the maintenance of epithelial barrier integrity, ultimately leading to dysbiosis and host metabolic disorders (Thaiss et al., 2014). Notably, bacterial functional activity and production of metabolites are also under the control of the host circadian clock. Indeed, microbial metabolites might act as key mediators of the modulation of host circadian rhythms by the microbiota, with potentially important consequences for host pathophysiological status. One such example is the modulation of hepatotoxicity after drug administration recently described by Gong et al. (2018). The authors reported that the gut microbiota mediates the diurnal variation in liver toxicity produced by acetaminophen, through the rhythmic production of the microbial metabolite 1-phenyl-1,2-propanedione that, by depleting hepatic reserve of the antioxidant glutathione at ZT12, enhances the risk of acute liver injury at this time point.

An extended description of the circadian regulation of the microbiota is not the focus of this review. However, for the purposes of evaluating the relations between host and microbes as a function of circadian cycles, it is interesting to note how the symbiotic community of host and microorganisms profoundly influences each other's activities and coordinates these activities to the day–night variations in the environment. It was recently shown how a metabolically favorable host response can drive a potentially lethal enteric bacterial pathogen to become a commensal microorganism and forsake virulence (Cadwell, 2018). Intriguingly, given the tight connection between circadian clock and metabolism, it is quite conceivable the possibility that the switch between pathogenicity and commensalism might be influenced by variation, as well as malfunctions, of the clock system in the host.

## Discussion and Future Directions

The past decades have brought important progress in our understanding of how the circadian system permeates all aspects of our physiology, including how our immune system endogenous clock ticks in time to confer levels of protection adequate to the risk of encounter with pathogens at specific times of the day. Altogether, examples described above highlight the importance of circadian rhythms in the host-pathogen interaction whereby daily fluctuations in the availability of resources and the nature of the immune response, and the pathogen itself, may influence microbial virulence, level of colonization and damage to the host, and alter the equilibrium between commensal and invading microorganisms. However, many aspects of the circadian regulation of immune and anti-microbial response remain unexplored. For instance, the antimicrobial effector functions of immune cells are strongly intertwined with the regulation of intracellular energy metabolism, but how the circadian clock participates in this regulation is still largely unknown. Also, how the circadian clock enables the precise integration of information, including neural, hormonal, and local inputs, across different immune cells has remained almost completely unexplored. Finally, whether the presence of a circadian rhythm in microbial species influences their colonization and virulence in human hosts is another fascinating topic that needs to be explored. Indeed, the answer to this question might have an impact on several different processes in which bacteria and fungi are directly involved, spanning from medical to agricultural and industrial areas of research and application. In human health, how a potential endogenous clock could influence microbial colonization and virulence in human hosts is tremendously important. Indeed, circadian changes not only influence the outcome of the host-pathogen interaction, but might also indicate the best time for intervention to potentiate the anti-microbial activities of an active immune system and to weaken the pathogen when its susceptibility is higher. Finally, a complete understanding of the daily rhythm of immune responses and the role of the circadian clock in the host response against pathogens might open a new prospect for correcting pathologies associated with aberrant immunity and inflammation.

### Circadian Medicine of Infections

The specific timing of drug delivery, or chronotherapy, is increasingly being recognized in clinical practice as the circadian clock affects the pharmacokinetics and pharmacodynamics of the drug, as well as the expression and/or activity of the target itself (Peeples, 2018). Examples of successful chronotherapy are already in place in clinical practice including, among others, the administration of the corticosteroid methylprednisolone for the treatment of arthritis and asthma, nonsteroidal anti-inflammatory drugs for rheumatoid arthritis and osteoarthritis, or cholesterol-lowering statins. A randomized trial comparing the protection obtained following influenza vaccination revealed that patients receiving the vaccine in the morning developed a greater antibody titer compared to the group vaccinated in the afternoon (Long et al., 2016). In oncology, several studies have shown how the chronomodulated administration of different combination chemotherapy significantly reduces side effects and impacts on survival (Levi et al., 2010). Notwithstanding the remarkable success of these examples and the positive data available, chronotherapy is still confined to a limited number of indications and a major conceptual advance is needed to extend its use that may potentially apply to any type of therapy.

The identification of pathways that link the circadian rhythms to infections in pre-clinical studies, followed by the development of reliable biomarkers that can confidently indicate the best timing for drug administration, might boost the development of a chronotherapeutic strategy that holds promises of major advances in our current therapeutic approach to diseases (Cederroth et al., 2019). In the context of infectious disease, a conceptual advance over the classical pharmacological drug-target paradigm is urgently required to optimize our current therapeutic approach and drive the therapy to infections according to the daily fluctuations of the host and the target, thus maximizing the safety and efficacy. The identification of circadian rhythms in microbial species, either endogenous or host-driven, driving their colonization and virulence in mammalian hosts, could substantially change the way we approach antibiotic treatments, revealing the time window of maximum susceptibility of targeted microbes in each tissue. This approach will likely bring us in an era of personalized circadian medicine, the appearance of which is creating the need to re-contextualize our thinking on the drug-target interaction. Indeed, circadian rhythms permeate all domains of life and regulate fundamental biological processes that reverberate in health and disease. It follows that the drug and the target are not static entities, but interact in an environment that changes along a circadian pattern, thus taking the classic concepts of pharmacokinetics and pharmacodynamics a step forward. A novel concept of pharmacological therapy that centers on the circadian rhythms of the host and the target can pave the way for novel therapeutic approaches that optimize the therapeutic effect while reducing the detrimental side effects.

## Author Contributions

CC and MMB wrote the paper with substantial contribution from all author listed.

### Conflict of Interest

The authors declare that the research was conducted in the absence of any commercial or financial relationships that could be construed as a potential conflict of interest.
